# Incorporation and Repair of Epigenetic Intermediates as Potential Chemotherapy Agents

**DOI:** 10.3390/molecules30153239

**Published:** 2025-08-01

**Authors:** Jason L. Herring, Mark L. Sowers, James W. Conrad, Linda C. Hackfeld, Bruce Chang-Gu, Rahul Dilawari, Lawrence C. Sowers

**Affiliations:** 1 Department of Pharmacology and Toxicology, University of Texas Medical Branch, 301 University Boulevard, Galveston, TX 77555, USA; 2 MD-PhD Combined Degree Program, University of Texas Medical Branch, 301 University Boulevard, Galveston, TX 77555, USA; 3 Department of Internal Medicine, University of Texas Medical Branch, 301 University Boulevard, Galveston, TX 77555, USA

**Keywords:** chemotherapy, DNA, glioblastoma, epigenetic intermediates, trifluorothymidine, TFT, 5-hydroxymethyuracil, 5HmdU, BER, SMUG1

## Abstract

The incorporation of nucleoside analogs into DNA by polymerases, followed by their removal through base excision repair (BER), represents a promising strategy for cancer chemotherapy. In this study, we investigated the incorporation and cytotoxic effects of several nucleoside analogs—some of which are epigenetic reprogramming intermediates—in the U87 glioblastoma cell line. We found that two analogs, 5-hydroxymethyl-2′-deoxyuridine (5HmdU) and trifluorothymidine (TFT), are both cytotoxic and are efficiently incorporated into genomic DNA. In contrast, the 5-carboxy analogs—5-carboxy-2′-deoxyuridine (5CadU) and 5-carboxycytidine (5CadC)—showed no cytotoxicity and were not incorporated into DNA. Interestingly, 5-hydroxymethyl-2′-deoxycytidine (5HmdC) was cytotoxic but was not directly incorporated into DNA. Instead, it was deaminated into 5HmdU, which was then incorporated and likely responsible for the observed toxicity. 5HmdU is actively removed from DNA through the BER pathways. In contrast, TFT remains stably incorporated and is neither excised by BER nor does it hydrolyze into 5CadU—a known substrate for the DNA glycosylase SMUG1. We also found that N^6^-benzyladenosine (BzAdo), an inhibitor of the enzyme 2′-deoxynucleoside 5′-phosphate N-hydrolase (DNPH1), enhances the cytotoxicity of 5HmdU. However, the thymidine phosphorylase inhibitor tipiracil hydrochloride (TPI) does not increase the cytotoxic effect of TFT in U87 cells. Together, these findings highlight 5HmdU and TFT as promising chemotherapeutic agents for glioblastoma, each with distinct mechanisms of action and cellular processing.

## 1. Introduction

The use of exogenous nucleoside analogs that are incorporated into DNA by polymerases and subsequently removed through the base excision repair (BER) pathway presents a promising strategy for cancer chemotherapy [1,2,3,4,5]. This approach leverages the cell’s natural repair mechanisms to selectively target and eliminate proliferating tumor cells.

In normal cells, DNA bases damaged by endogenous oxidation and hydrolysis are routinely removed by the BER pathway [6,7]. More recently, it has been discovered that several endogenous DNA bases can also undergo enzymatic modification through oxidation and hydrolysis as part of epigenetic reprogramming. These modified bases, such as 5-formylcytosine and 5-carboxycytosine, are also substrates for BER [8].

In a recent study, we demonstrated that oxidized and deaminated derivatives of 5-methylcytosine—including 5-formylcytosine, 5-carboxycytosine, 5-hydroxymethyluracil, 5-formyluracil, and 5-carboxyuracil—are efficiently recognized and excised by DNA glycosylases [9].

During nucleotide excision repair or homologous recombination, DNA containing both normal and modified bases can be degraded to monophosphates and recycled for new DNA synthesis [10,11]. However, if the nucleotide pool is not adequately purged of these modified bases—especially those involved in epigenetic regulation—their random reincorporation could disrupt normal gene expression. For instance, the reuse of 5-methyl-2′-deoxycytidine-5′-monophosphate is minimized by its enzymatic deamination to dTMP [12].

While the reutilization of modified nucleosides poses a risk to normal cells, it can be exploited therapeutically. Exogenous modified bases, when incorporated during DNA replication and subsequently excised by glycosylases, may induce cytotoxicity in rapidly dividing tumor cells. One example is the incorporation of 5-hydroxymethyl-2′-deoxyuridine (5HmdU), which has been proposed as a treatment for leukemia [2].

**Figure 1:**
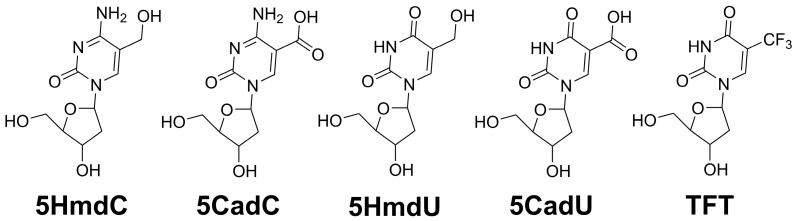
Structures of the nucleoside analogs examined in this study.

In this study, we evaluate the cytotoxic potential of several 2′-deoxynucleosides—5HmdU, 5HmdC, 5CadU, 5CadC—in the glioblastoma cell line U87. We also include TFT (also known as trifluridine), a compound known to hydrolyze to 5CadU [13]. TFT has demonstrated anticancer activity in various tumor types [14] and has been proposed as a potential therapeutic agent for glioblastoma [15]. Our goal was to determine whether the hydrolysis of TFT to 5CadU—either before or after its incorporation into DNA—contributes to its cytotoxic effects. The chemical structures of these modified nucleosides are shown in Figure 1.

## 2. Results

**Figure 2:**
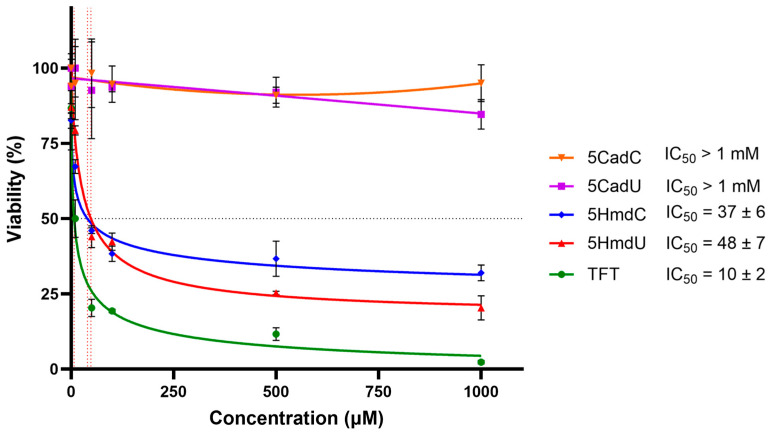
The nucleoside analogs TFT, 5HmdU, and 5HmdC are toxic to U87 cells in culture whereas 5CadC and 5CadU are not. Cell viability was assessed by the MTT assay at 3 days using 2 × 10^3^ cells. Decreasing cell viability is seen with increasing nucleoside concentration with active nucleosides. The nucleoside analogs 5CadU and 5CadC are not toxic in this assay. Each experiment was run in triplicate. Error bars represent the standard deviation of three determinations.

U87 glioblastoma cells were treated with increasing concentrations of various nucleoside analogs for three days, and cytotoxicity was measured using the MTT assay (Figure 2). The results showed that 5HmdU, 5HmdC, and TFT significantly reduced cell viability, indicating cytotoxic effects. In contrast, 5CadU and 5CadC did not exhibit cytotoxicity under the same conditions. Among the active compounds, TFT was the most potent, with an IC_50_ of approximately 10 µM. 5HmdU and 5HmdC showed moderate cytotoxicity, with IC_50_ values of 48 µM and 37 µM, respectively.

**Figure 3:**
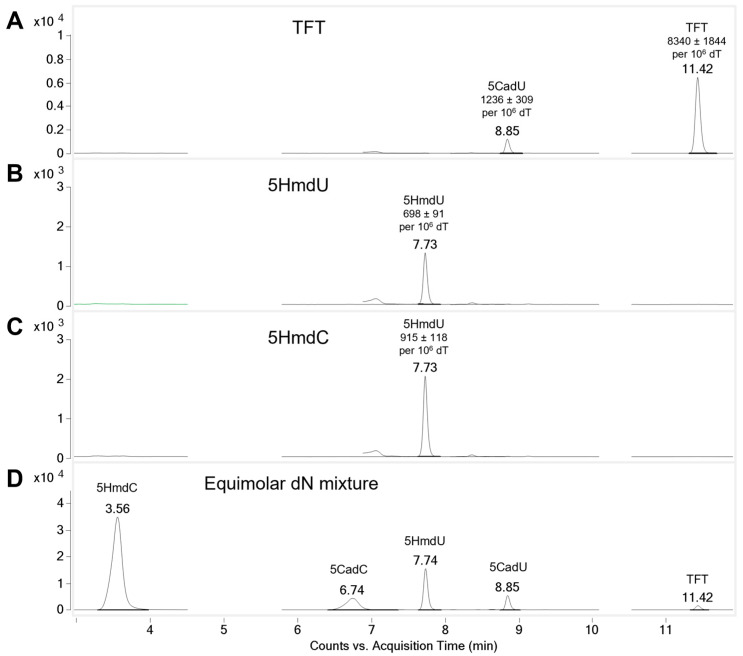
TFT and 5HmdU are incorporated into DNA. The incorporation of nucleoside analogs into DNA of cells in culture was measured by LC-MS/MS. Nucleoside analogs TFT and 5HmdU are incorporated into DNA while 5CadU, 5CadC, and 5HmdC are not incorporated. (**A**) Approximately 8340 ± 1844 TFT per 10^6^ dT molecules were incorporated in 4 h at 5 µM TFT. Approximately 12.9% TFT is hydrolyzed to 5CadU (1236 ± 309 per 10^6^ dT) during DNA digestion. (**B**) Approximately 698 ± 91 5HmdU per 10^6^ dT are incorporated into DNA. (**C**) 5HmdC is not incorporated into DNA, but, its enzymatic deamination product, 5HmdU is observed in DNA at 915 ± 118 per 10^6^ dT. (**D**) LC-MS/MS separation of nucleoside standards. In each experiment, 2 × 10^6^ cells were used. The average numbers of nucleosides and standard deviations were determined from three experimental replicates.

To determine whether the nucleoside analogs are incorporated into genomic DNA, U87 cells were treated with 5 µM of each compound for 4 h. Genomic DNA was then extracted, enzymatically digested, and analyzed by LC-MS/MS. No incorporation of 5CadU or 5CadC was detected (Figure S1). In contrast, cells treated with 5HmdU showed a clear peak corresponding to 5HmdU in the DNA hydrolysate (Figure 3), confirming its incorporation. Interestingly, 5HmdC was not directly incorporated; instead, a peak corresponding to 5HmdU was observed, suggesting that 5HmdC is deaminated to 5HmdU prior to incorporation. For cells treated with TFT, peaks corresponding to both TFT and its hydrolysis product, 5CadU, were detected in the DNA, indicating incorporation of both forms (Figure 3).

To quantify the incorporation of nucleoside analogs into genomic DNA, relative peak areas from LC-MS/MS analysis were normalized to thymidine levels. All experiments were performed in triplicate. After 4 h of treatment, cells exposed to 5HmdU incorporated an average of 698 ± 91 molecules per 10^6^ thymidine. Although 5HmdC itself was not detected in the DNA, cells treated with 5HmdC showed the incorporation of 915 ± 118 5HmdU molecules per 10^6^ thymidine, suggesting that 5HmdC is deaminated to 5HmdU before incorporation. TFT exhibited the highest incorporation, with 8340 ± 1844 analogs per 10^6^ dT. Additionally, its hydrolysis product, 5CadU, accounted for approximately 12.9% of the total TFT-related signal detected in the DNA hydrolysate (Figure 3).

To assess the pH-dependent hydrolysis of TFT to 5CadU, we monitored TFT concentrations over time in phosphate-buffered aqueous solutions at 37 °C (Figure S2). At physiological pH (7.4), which mimics cell culture conditions, TFT hydrolyzed with a half-life of 34.4 h. In contrast, at pH 8.0—the pH of the enzymatic DNA hydrolysis buffer—the hydrolysis proceeded more rapidly, with a reduced half-life of 13.5 h.

**Figure 4:**
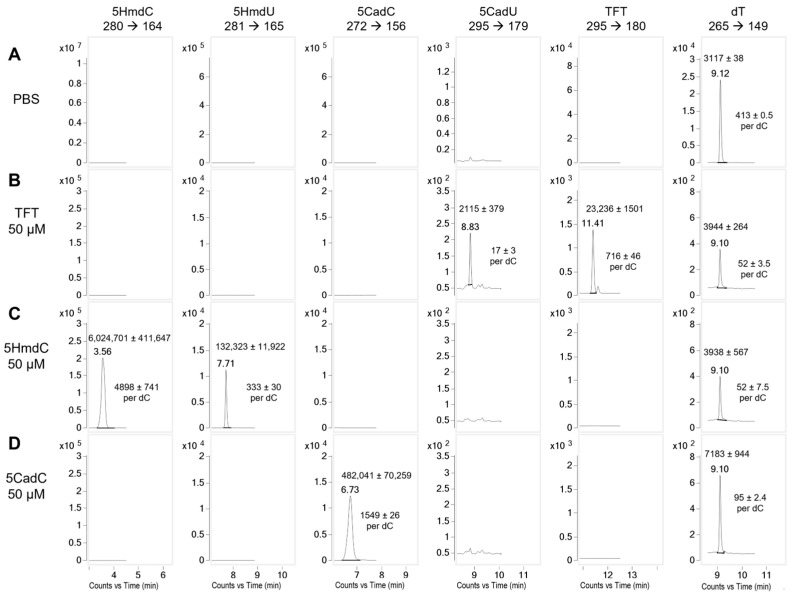
Nucleoside analogs can be modified in the cytoplasm. (**A**) Control (PBS) showing that nucleoside analogs are not detectable in the cytoplasm of untreated cells. (**B**) When TFT is added to cultured cells at 50 µM, both TFT and 5CadU can be seen. However, only 2.3% TFT hydrolyzes to 5CadU in that time. (**C**) When 5HmdC is added to media, both 5HmdC and 5HmdU are observed. At 1 h, approximately 6.3% of the 5HmdC has been deaminated to 5HmdU. (**D**) 5CadC is not deaminated to 5CadU at 1 h. In each experiment, 1.7 × 10^6^ cells were used. The average number of nucleosides and standard deviations were determined from three separate injections onto the LC-MS/MS.

To evaluate the stability of the nucleoside analogs prior to DNA incorporation, each compound was incubated in cell culture media with U87 cells for 1 h. Following incubation, cytoplasm was collected and analyzed by LC-MS/MS (Figure 4). The hydrolysis product of TFT, 5CadU, was detected at 2.3%, closely aligning with the 2.5% hydrolysis predicted from its known rate in buffered solutions. In contrast, although 5HmdC is stable in aqueous solution, a peak corresponding to its deamination product, 5HmdU, was observed. This accounted for 6.3% of the combined 5HmdU and 5HmdC signal, suggesting partial deamination of 5HmdC under cell culture conditions at 1 h.

**Figure 5:**
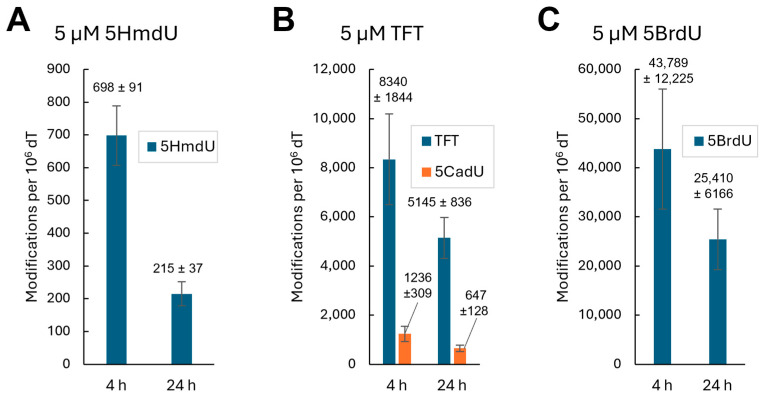
5HmdU is repaired from DNA but TFT is not. (**A**) The amount of 5HmdU in the DNA is diminished from 4 to 28 h to 30.8% of its initial value by replication-dilution as well as from BER. (**B**,**C**) The TFT and 5BrdU fall to 61.7% and 58.0% of their initial values from 4 to 28 h due to replication-dilution. (**B**) The amount of 5CadU arising from TFT hydrolysis is 12.9% at 4 h and 11.1% at 28 h and is accounted for by hydrolysis during DNA digestion. In each experiment, 2 × 10^6^ cells were used. The average numbers of nucleosides and standard deviations were determined from three separate injections onto the LC-MS/MS from three experimental replicates.

To investigate whether nucleoside analogs are actively removed from DNA by base excision repair, U87 cells were first incubated with 5 µM of 5HmdU, TFT, or 5-bromo-2′-deoxyuridine (5BrdU) for 4 h (Figure 5). Following this treatment, cells were transferred to analog-free media and incubated for an additional 24 h. 5BrdU served as a control to account for passive decrease in analogs due to DNA replication and dilution. After 24 h of cell replication, the amount of 5BrdU in DNA decreased to 58.0% of the initial level. In comparison, 5HmdU levels dropped more substantially, to 30.8%, suggesting that its removal is not solely due to replication dilution but also involves active excision by base excision repair mechanisms. In contrast to 5HmdU, TFT levels in DNA decreased to 61.7% after 24 h, which is comparable to the 58.0% decrease observed for 5BrdU. This similarity suggests that TFT is not actively removed by DNA repair mechanisms and is instead diluted through DNA replication.

The hydrolysis product of TFT, 5CadU, accounted for 12.9% of the total TFT-related signal in DNA incubated with TFT for 4 h. During enzymatic digestion of DNA, which involves incubation at pH 8 for 3 h, 5CadU formation is expected due to hydrolysis. Based upon the hydrolysis rate in solution, this process would be predicted to yield approximately 15.3% 5CadU—closely matching the observed 12.9%. After an additional 24 h incubation, the measured 5CadU level slightly decreased to 11.1%, indicating minimal further hydrolysis. Notably, if TFT were freely hydrolyzing in solution at pH 7.4 for 24 h, 41% would be expected to convert to 5CadU. Therefore, the relatively stable 5CadU levels (12.9% vs. 11.1%) suggest that most of the observed 5CadU arises during enzymatic digestion, not from in-cell or in-DNA hydrolysis.

**Figure 6:**
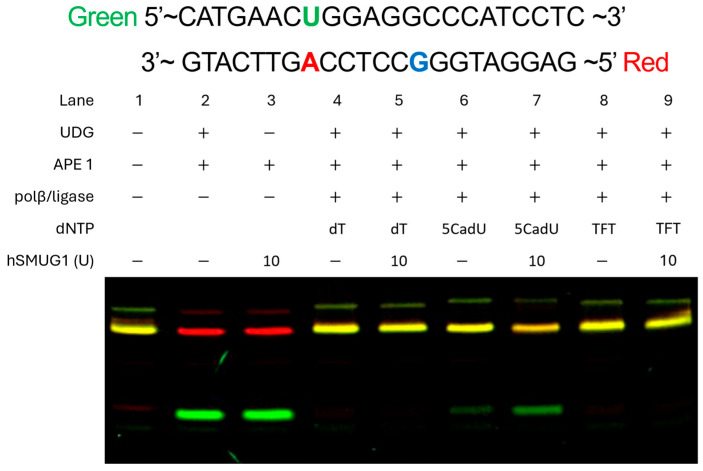
Repair of duplex DNA containing a TFT:A base pair or a 5CadU:A base pair. A 79 base pair duplex (U:A) was incubated with uracil DNA-glycosylase (UDG) in CutSmart^TM^ buffer (New England Biolabs, Ipswich, MA, USA), 5 U of apurinic endonuclease (APE1), 6 pmol DNA polβ, 5 U *E. coli* ligase, 26 mM NAD^+^ and 20 mM of each indicated dNTP in 12.5 µL at 37 °C for 1 h. SMUG1 (10 U, New England Biolabs) was added and reactions were incubated at 37 °C for 1 h. Samples were run on denaturing 15% PAGE. The U:A base pair is processed by UDG and APE1 creating a repair gap. This gap is filled with DNA polβ and a modified dNTP (5CadU-TP or TFT-TP) and ligated with DNA ligase. The 5CadU, but not TFT, is cleaved by SMUG1.

To investigate whether TFT and 5CadU are substrates for glycosylase-mediated base excision, synthetic oligonucleotide duplexes were constructed with each analog paired opposite adenine (Figure 6). These duplexes were then incubated with human SMUG1 glycosylase. The duplex containing 5CadU:A was cleaved by SMUG1, indicating the recognition and excision of 5CadU. In contrast, the TFT:A duplex was not cleaved, suggesting that TFT is not a substrate for SMUG1 under these conditions.

**Figure 7:**
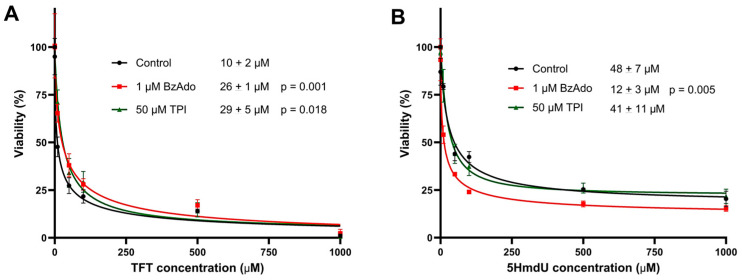
The toxicity of 5HmdU is increased by BzAdo but the toxicity of TFT is not increased by TPI. The 3-day toxicity of TFT (**A**) and 5HmdU (**B**) were examined in U87 cells exposed to 1 µM BzAdo, 50 µM TPI or no inhibitor. The IC_50_ of 5HmdU decreased 4-fold from 48 ± 7 µM to 12 ± 3 µM with BzAdo. In each experiment, 2.2 × 10^3^ cells were used. GraphPad Prism software (Version 10.5.0) was employed to calculate IC_50_ values and error bars determined from 3 experimental replicates.

To evaluate the influence of potential inhibitors on the cytotoxicity of nucleoside analogs, cytotoxicity assays were performed in the presence and absence of two inhibitors: 50 µM tipiracil hydrochloride (TPI) and 1 µM N^6^-benzyladenosine (BzAdo) (Figure 7). In the presence of either TPI or BzAdo, the cytotoxicity of TFT was slightly reduced, as indicated by modest increases in its IC_50_. In contrast, the cytotoxicity of 5HmdU was significantly enhanced by BzAdo, with the IC_50_ decreasing from approximately 48 µM to 12 µM. This suggests that BzAdo may inhibit the hydrolysis of 5HmdUMP within cells.

**Figure 8:**
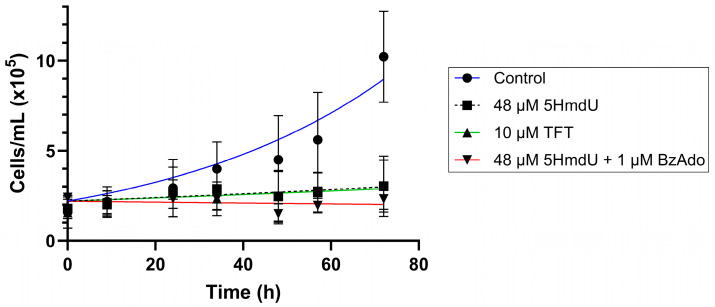
U87 proliferation in the presence of the nucleoside inhibitors as a function of time is in accord the cytotoxicity assays. Approximately 2.2 × 10^4^ cells in 1 mL were seeded per well into 7 12-well plates per experiment. After allowing cells to attach overnight, cells were treated with 48 µM 5HmdU, 10 µM TFT or 48 µM 5HmdU plus 1 µM BzAdo before placing in 10% CO_2_ incubator. A single plate was removed and cells counted by Trypan Blue exclusion at 0, 9, 24, 34, 48, 57 and 72 h after adding analogs. Error bars represent triplicate cell counts from four experimental replicates.

In Figure 8, we show the proliferation of U87 cells in the presence of TFT, 5HmdU, and inhibitors of 5HmdU degradation.

We observe that when at their IC_50_ concentrations, both TFT and 5HmdU inhibit the proliferation of U87 cells in culture. When BzAdo, an inhibitor of 5HmdUMP hydrolysis, is present, a decrease in cell numbers is observed.

To examine the mechanisms of cell death induced by TFT and 5HmdU, we performed two assays, a lactate dehydrogenase (LDH) release assay and a caspase-3 activation assay (Figures S3 and S4). We observed no increase in the LDH activity released by cells when treated with TFT and 5HmdU at their respective IC_50_’s. In contrast, we found that both 5HmdU and TFT induced caspase-3 activity. The magnitude of the increase in caspase-3 activity is higher with 5HmdU and it appears earlier (Figure S4).

Finally, to compare the toxicity of TFT and 5HmdU in normal cells, we examined survival of normal human astrocytes (NHA) in the presence of these analogs. When grown under similar conditions of media and CO_2_ concentration, the IC_50_ of 5HmdU was found to be 143 ± 8 µM and that of TFT to be 784 ± 96 µM, more than 7 times higher in NHA than in the U87 cell line. Remarkably, 5HmdC was observed to be nontoxic to normal human astrocytes (Figure S5).

## 3. Discussion

Glioblastoma is an aggressive and fatal brain tumor that remains largely unresponsive to current therapies, with median survival following diagnosis rarely exceeding 18 months [16,17]. As a result, there is an urgent need for novel treatment strategies. In this study, we investigate the potential of nucleoside analogs—specifically those that function as epigenetic intermediates—as chemotherapeutic agents (Figure 1). These compounds are designed to be incorporated into newly synthesized DNA, where they may disrupt replication or trigger base excision repair, ultimately enhancing cytotoxicity. Prior research has shown that the incorporation and subsequent excision of 2′-deoxyuridine, induced by agents such as 5-fluoro-2′-deoxyuridine (5FdU) or antifolates, can be an effective chemotherapeutic approach [1,3].

Within DNA, 5-methyl-2′-deoxycytidine (5mdC) can undergo enzymatic oxidation to form 5HmdC, 5-formyl-2′-deoxycytidine (5FodC), and 5CadC. These oxidized cytosine derivatives can potentially be deaminated to their corresponding uracil analogs: 5HmdU, 5-formyl-2′-deoxyuridine (5FodU), and 5CadU, respectively [8,9]. While the fate of these modified nucleosides in DNA remains unclear, several have been shown to be substrates for base excision repair [9]. In this study, we did not include the 5-formyl derivatives (5FodC and 5FodU) due to the high reactivity of the formyl group, which may lead to cross-linking with amino acids [18]. However, we included TFT, a nucleoside analog that readily hydrolyzes to 5CadU. TFT is a component of the chemotherapeutic formulation TAS-102, which also includes thymidine phosphorylase inhibitor (TPI) to prevent degradation of TFT to its free base [19].

In the initial set of experiments, we evaluated the cytotoxicity of five 2′-deoxynucleoside analogs: 5HmdU, 5HmdC, 5CadU, 5CadC, and TFT. As shown in Figure 2, these compounds displayed a range of cytotoxic effects. The analogs 5CadU and 5CadC showed no detectable toxicity. In contrast, 5HmdU, 5HmdC, and TFT were cytotoxic, with IC_50_ values ranging from 10 to 48 µM. Among them, TFT was the most potent, while 5HmdU and 5HmdC exhibited similar levels of cytotoxicity.

Next, we examined whether the nucleoside analogs could be incorporated into the DNA of replicating U87 cells. A concentration of 5 µM was used—approximately half the IC_50_ of TFT—to avoid complete inhibition of DNA replication. After a 4 h incubation with each analog, genomic DNA was extracted, enzymatically digested, and analyzed by LC-MS/MS (Figure 3). No incorporation of 5CadU or 5CadC was detected (Figure S1), consistent with their lack of cytotoxicity.

We next examined the potential metabolism or conversion of the nucleoside analogs by incubating them at 50 µM in cell culture media with U87 cells for 1 h (Figure 4). No changes were observed in 5HmdU levels during this period, indicating its stability. In contrast, TFT underwent partial hydrolysis to 5CadU, with 2.3% conversion observed—closely matching the 2.5% expected from its hydrolysis rate in phosphate buffer. Since 5CadU is not incorporated into DNA, this slow hydrolysis of TFT in the media or cytoplasm is unlikely to significantly contribute to its cytotoxicity.

Substantial incorporation of TFT into DNA was observed (Figure 3). 5CadU was also detected in the DNA digest of TFT-treated cells; however, this is consistent with partial hydrolysis of TFT during the 3 h enzymatic digestion process. 5HmdU was incorporated into DNA without modification. In contrast, cells treated with 5HmdC showed incorporation of only 5HmdU, indicating that 5HmdC is enzymatically deaminated prior to incorporation. Notably, all three nucleoside analogs that exhibited cytotoxicity—TFT, 5HmdU, and 5HmdC—were incorporated into DNA, although 5HmdC was incorporated in the form of 5HmdU.

We found that 5HmdC undergoes deamination to 5HmdU in the cytoplasm when incubated under physiological conditions with U87 cells. After 1 h, approximately 6.3% of the 5HmdC was converted to 5HmdU. Consistent with this, we observed that 5HmdU—but not 5HmdC—is incorporated into DNA. For nucleoside analogs to be incorporated into DNA, they must first be phosphorylated to their mono-, di-, and triphosphate forms to serve as substrates for DNA polymerases. In contrast, 5CadC was neither incorporated into DNA nor deaminated in the U87 cell line.

Previous studies have shown that 2′-deoxycytidine (dC) and 5-methyl-2′-deoxycytidine (5mdC) are rapidly deaminated but are inefficiently phosphorylated to their monophosphate forms [12]. Our findings suggest that 5HmdC behaves similarly: it is readily deaminated to 5HmdU but is not efficiently phosphorylated, which likely explains its absence from DNA.

There are potentially three deaminases that might account for the deamination of 5HmdC in glioblastoma: deoxycytidine deaminase, dCDA [20], cytidine deaminase, CDA [21] and dCMP deaminase, DCTD [22]. In experiments with recombinant CDA isolated from peripheral blood polymorphonuclear leukocytes [4], it was shown that 5HmdC is deaminated but 5CadC is not. The similar substrate specificity observed in our study with prior results obtained with recombinant CDA suggests that CDA may be the primary deaminase for 5HmdC in U87 cells. The absence of toxicity of 5HmdC toward normal human astrocytes suggest that CDA levels may vary from one cell type to another and potentially exploited for chemotherapy development.

The mechanism of action of TFT is not fully understood. Originally synthesized by Heidelberger et al. in 1964 [23], TFT is efficiently transported into cells via the equilibrative nucleoside transporters ENT1 and ENT2 [19]. Once inside the cell, TFT is rapidly phosphorylated by thymidine kinase [24]. As a monophosphate, TFT can inhibit thymidylate synthase, a key enzyme in nucleotide biosynthesis. Additionally, TFT is known to be incorporated into the DNA of replicating cells [25]. In its triphosphate form (TFT-TP), it competes with the natural nucleotide TTP for incorporation opposite adenine during DNA synthesis [26]. Although TFT does not cause chain termination, its incorporation significantly slows DNA synthesis—by approximately 60% [27,28]. The *K_m_* of TFT-TP for HeLa cell DNA polymerase is about four times higher than that of TTP, indicating lower binding affinity [28].

TFT incorporated into DNA may undergo hydrolysis to 5CadU, potentially altering its recognition by DNA repair enzymes. While TFT mispaired with guanine can be excised by the glycosylases Methyl-CpG binding domain protein 4 (MBD4) or thymine DNA glycosylase (TDG), it is not a substrate for either enzyme when correctly paired with adenine [29]. Given that the half-life for TFT hydrolysis to 5CadU is approximately 34.4 h at pH 7.4—shorter than the 72 h duration used in cytotoxicity assays—we considered the possibility that TFT is initially incorporated into DNA and subsequently hydrolyzed to 5CadU. This conversion could create a lesion recognizable by SMUG1 glycosylase. Supporting this hypothesis, we previously demonstrated that 5CadU is a strong substrate for SMUG1 when paired with either adenine or guanine [9].

To compare the enzymatic excision of TFT and 5CadU by SMUG1, we synthesized DNA templates containing either TFT or 5CadU paired with adenine using a recently developed base excision repair method (Figure 6), incorporating TFT-TP and 5CadU-TP [30]. These duplexes were then tested as substrates for SMUG1 glycosylase. We observed that SMUG1 efficiently excised 5CadU when paired with adenine, but did not excise TFT from the TFT:A pair. These results suggest that if TFT undergoes hydrolysis after incorporation into DNA, the resulting 5CadU could become a substrate for SMUG1, potentially contributing to TFT’s cytotoxic effects.

To investigate whether TFT undergoes hydrolysis while incorporated in DNA, we compared the levels of 5CadU in DNA at 4 and 28 h following TFT treatment. The levels remained essentially unchanged (12.9% at 4 h vs. 11.1% at 28 h), indicating that TFT is stable once incorporated into DNA and is not hydrolyzed in situ. The 5CadU detected likely arises during the enzymatic digestion process, which involves incubation at pH 8.0, a condition known to promote TFT hydrolysis. Since TFT is not a substrate for SMUG1 glycosylase (Figure 6), and its levels in DNA do not decrease over time—as observed with 5HmdU—it is unlikely that base excision repair contributes to its cytotoxicity.

Our nucleoside incorporation studies reveal TFT is incorporated into DNA at a rate 13.7 times higher than 5HmdU. Once incorporated, TFT remains in the DNA and is not subject to excision repair. In contrast, 5HmdU is actively removed by base excision repair. This difference is evident when comparing DNA retention levels after 24 h: TFT and 5-bromo-2′-deoxyuridine (5BrdU) levels decreased to 61.7% and 58.0%, respectively, due to replication-dilution. However, 5HmdU levels drop more substantially to 30.8%, reflecting the combined effects of replication-dilution and excision repair. The excision of 5HmdU has been previously reported in other cell lines [31,32,33]. We recently confirmed that 5HmdU paired with adenine is a substrate for SMUG1 glycosylase, supporting its active removal from DNA [9].

The mechanisms of cell death induced by TFT and 5HmdU are likely complex and different. In previous studies, the capacity of 5HmdU to trigger apoptosis by inducing base excision repair has been highlighted [34,35,36], and this is consistent with data reported here showing apoptosis in the U87 cell line. In previous studies, the toxicity of TFT has been attributed to both caspase-dependent and independent pathways [37,38,39]. Like 5HmdU, TFT is incorporated into the DNA of replicating cells. However, our evidence suggests it is not a substrate for excision by the base excision repair pathway. Instead, TFT’s ability to slow DNA replication, leading to replication stress likely underlies its cytotoxic effects. This is supported by previous findings showing that TFT treatment activates Chk1 and induces H2AX phosphorylation [40,41]. We show here that both TFT and 5HmdU activate caspase-3 and induce apoptosis.

In follow-up studies (Figure 7), we investigated whether inhibiting the degradation of nucleoside analogs could enhance their cytotoxicity. TFT is known to be degraded by thymidine phosphorylase [42], while 5HmdU monophosphate can be hydrolyzed by 2′-deoxynucleoside 5′-phosphate N-hydrolase (DNPH1) [43]. These degradation pathways can be inhibited by specific compounds: TFT degradation is blocked by the thymidine phosphorylase inhibitor, TPI [44], and 5HmdU degradation is inhibited by BzAdo [36]. Interestingly, co-treatment with 50 µM TPI did not enhance TFT cytotoxicity. In contrast, co-treatment with 1 µM BzAdo significantly increased the cytotoxicity of 5HmdU, reducing its IC_50_ from 48 ± 7 µM to 12 ± 3 µM. These findings suggest that TFT hydrolysis may not be significant in U87 cells; however, 5HmdUMP hydrolysis is significant but can be inhibited. Data obtained in 3-day toxicity studies are recapitulated in proliferation studies (Figure 8).

In summary, TFT is incorporated intact into DNA, and its cytotoxic effects are evident before significant hydrolysis to 5CadU occurs. The primary mechanism of TFT’s cytotoxicity appears to be interference with DNA replication and activation of the DNA damage response. In contrast, 5HmdU is also incorporated into DNA, but its cytotoxicity is partly driven by subsequent excision via base excision repair. Notably, the cytotoxicity of 5HmdU—but not TFT—can be enhanced by inhibiting its degradation in U87 cells. These findings support further investigation of both TFT and 5HmdU, and possibly 5HmdC, as potential chemotherapeutic agents for glioblastoma.

## 4. Materials and Methods

5-Carboxy-2′-deoxyuridine (5CadU, LS Hiconc 9-16-24) and 5-(Hydroxymethyl)-2′-deoxyuridine (5HmdU, LS 06-22-91) were synthesized in the lab as previously reported [45,46]. Commercially available nucleosides were purchased as follows: Trifluorothymidine (TFT, Biosynth NT04884, Staad, Switzerland), 5-carboxy-2′-deoxycytidine (5CadC, Jena Bioscience N-1091-5, Jena, Germany), 2′-deoxythymidine (dT, Chem-Impex International A0000-03-06, Wood Dale, IL, USA), 5-(Hydroxymethyl)-2′-deoxycytidine (5HmdC, Cayman Chemical 18162, Ann Arbor, MI, USA), N^6^-Benzyladenosine (BzAdo, MedChemExpress HY-N7844, Monmouth Junction, NJ, USA), Tipiracil (TPI, Sigma SML1552-10MG, Burlington, MA, USA) and 5-bromo-2′-deoxyuridine (5BrdU, Sigma B9285-250MG, Burlington, MA, USA). Benzonase was obtained from Sigma (E1014, Burlington, MA, USA). Proteinase K (RP107B-1) and RNase A (1007885) were purchased from Qiagen (Hilden, Germany). Bacterial Alkaline Phosphatase (BAP) was purchased from Invitrogen (18011015, Waltham, MA, USA). Phenol/chloroform/isoamyl alcohol (25:24:1) was from Fisher Scientific (BP-1752I-100, Waltham, MA, USA).

### 4.1. Determination of Rates of Hydrolysis of TFT in Phosphate Buffer, pH 7.4 and 8.0

The rates of hydrolysis of TFT in buffered solution were measured with an HPLC coupled to a photodiode array detector (PDA). A solution of TFT (200 µM) was prepared containing an equimolar amount of 5-bromo-2′-deoxuridine as an internal standard. Solutions were maintained at 37 °C and aliquots (5 µL) were taken at measured intervals for HPLC analysis. Rates constants were determined by measuring relative peak sizes as a function of time in phosphate buffer (66 mM) at pH 7.4 and 8.0. Equations used for these determinations can be found in the Supporting Information section.

### 4.2. Cell Culture Conditions

U87 cells (ATCC) were propagated in high glucose DMEM supplemented with 10% fetal bovine serum (FBS) and 1% antibiotic/antimycotic mixture (Penicillin, Streptomycin and Amphotercin B, Corning 30-004-CI, Corning, NY, USA). A humid 37 °C incubator containing a 10% CO_2_ atmosphere was used. For toxicity assays, 2 × 10^3^ cells in 90 µL was added to each well on 96-well plates and allowed to attach for 16–18 h.

### 4.3. Determination of IC_50_ Values

The IC_50_ values were determined using the MTT assay in 96-well plates. Cells were incubated in DMEM media for 72 h, and the fraction of viable cells was measured using the MTT assay. GraphPad Prism (Version 10.5.0) software was employed to calculate IC_50_ values, which correspond to the concentration of nucleoside analog required to reduce the number of viable cells by 50%.

### 4.4. Measurement of Incorporation and Persistence of 2′-Deoxynucleosides in Genomic DNA

U87 cells (2 × 10^6^) were treated with 5 µM nucleoside analog in high glucose DMEM at 37 °C and incubated for 4 h. The media was subsequently removed, and the cells were washed with phosphate-buffer saline (PBS), pelleted, and the cell pellet was frozen for DNA extraction. Another plate of cells was incubated for 4 h with the nucleoside analog, washed with PBS, replated in fresh media without the analog, and returned to the incubator. After an additional 24 h, cells were harvested, treated with trypsin, washed with PBS, and pelleted. The cell pellets were frozen for DNA extraction and digestion.

### 4.5. DNA Extraction and Enzymatic Digestion

Pellets from 2–3 × 10^6^ cells were lysed in 270 µL lysis buffer (100 mM Tris pH 8.5, 200 mM NaCl, 5 mM EDTA, 0.2% SDS, 150 µg/mL Proteinase K, and 370 µg/mL RNase A) and incubated for 3 h at 37 °C. A solution of 8 M ammonium acetate was added to a final concentration of 1 M. DNA was extracted with phenol/chloroform isoamyl alcohol (25:24:1) and vortexed. The emulsion was centrifuged at maximum speed for 5 min, and the top layer was removed. DNA was precipitated with 3 volumes of cold absolute ethanol and collected by centrifugation. The DNA pellet was washed with ice-cold 70% ethanol, air-dried, and resuspended in water at 4 °C.

DNA was enzymatically digested to 2′-deoxynucleosides using a previously published method with minor modifications [47]. The DNA concentration was measured by UV on a nanophotometer (Implen, Munich, Germany). Approximately 5 µg of DNA and 5 µL of digestion mixture were added to a final volume of 25 µL water and incubated at 37 °C in a thermocycler (BioRad, Hercules, CA, USA) for approximately 4 h. Each digestion mixture contained 250 mM Tris pH 8, 5 mM MgCl_2_, 0.5 mg/mL BSA, 1 unit/µL Benzonase recombinant endonuclease, and 0.1 units/µL bacterial alkaline phosphatase (BAP). Liberated 2′-deoxynucleosides were fractionated from the crude digest using a spin filter (10 kDa, Millipore, Burlington, MA, USA).

The crude DNA digest plus 70 µL water was placed in the filter and spun at 16,000× *g* for 1 min. Approximately 200 µL water was added to the spin filter to dilute the remaining retentate, and the filter was spun at 16,000× *g* for 5 min. The eluate was concentrated under reduced pressure in a SpeedVac to approximately 40 µL. Approximately 8 µL was injected onto the LC-MS/MS.

### 4.6. LC-MS/MS Analysis

For LC-MS/MS analysis, 8 µL of the DNA digestion products was injected onto an Agilent 1260 Infinity LC system coupled to an Agilent 6490 triple quadrupole mass spectrometer. Separation was achieved using a Waters HSS T3 column (2.1 × 150 mm, 3.5 µm) with the following gradient separation at 350 µL/min: 0% B from 0 to 2 min, 0–20% B from 2 to 10 min, 20–80% from 10 to 12.5 min with a 1.5 min hold, and then back to 0% B for 10 min. Solvent A was water with 0.1% formic acid, and solvent B was acetonitrile (ACN) with 0.1% formic acid. Transitions of 265 *m*/*z* > 139 *m*/*z* for 2′-deoxythymidine (dT) (positive ion), 295 > 179 for 5CadU (positive ion), 281 > 165 for 5HmdU (positive ion), 295 > 180 for TFT (negative ion), and 331 > 215 for 5BrdU (positive ion) were monitored. Peak areas of extracted ion chromatograms were measured and used to determine concentrations of the 2′-deoxynucleosides.

Peak areas of the modified 2′-deoxynucleosides were converted to moles using standard curves and the Agilent Mass Hunter software (Version 12.0). Levels of the modified 2′-deoxynucleosides in DNA were then normalized relative to the thymidine peak for each injection and expressed as modifications per 10^6^ thymidine. Error bars were generated from triplicate LC-MS/MS injections of three experimental replicates.

### 4.7. Measurement of 2′-Deoxynucleosides in the Cytoplasm

Approximately 1.7 × 10^6^ U87 cells were exposed to 50 µM of the nucleoside analog for 1 h in high glucose DMEM. After 1 h, the media was removed, and cells were washed with PBS before trypsinization and collection. Cells were homogenized by swelling in ice-cold Milli-Q water prior to passing the homogenate through a 25-gauge needle 2 to 3 times. The soluble cytoplasmic fraction and insoluble nuclear fractions were separated by centrifugation (1000× *g*). The cytoplasmic fraction was cleared by centrifugation (max) and spin-filtered (3 kDa) prior to concentration under vacuum. The cytoplasmic fractions were examined by LC-MS/MS analysis.

### 4.8. Incorporation of TFT and 5CadUTP into a 79-Base Pair Oligonucleotide and Cleavage by SMUG1 Endonuclease

A 79-base pair DNA duplex was prepared containing a uracil-adenine base pair [31]. The duplex was incubated with uracil DNA glycosylase to create a gap in the DNA, which was subsequently cleaved with AP-endonuclease. Gap filling was then initiated using the corresponding dNTP and DNA polymerase, and the repair gap was closed by DNA ligase. Oligonucleotides were subsequently incubated with SMUG1.

### 4.9. Examination of Cell Death Pathways

LDH Assay. The activation of cell lytic pathways was evaluated with LDH Assay Kit (Abcam, cat# ab197000, Cambridge, UK) according to manufacturer’s protocol [48]. In brief, culture media was collected 24 h after exposure to TFT (10 µM) and 5HmdU (48 µM). An aliquot of culture media (50 µL) was mixed with 50 µL of reaction mix (45.5 µL LDH Assay Buffer, 2.5 µL PicoProbe, and 2 µL LDH Substrate Mix). NADH standards (0–500 pmol/well) were mixed with 50 µL of the reaction mix. Fluorescence was measured at regular intervals at Ex/Em = 535/595 nm for 30 min. LDH activity in the test samples were calculated as:

$$LDH\;Activity=\left(\frac{B}{∆T\times 50\;µL}\right)$$

where B is the NADH amount generated within ∆T and ∆T is the reaction time. One unit is the amount of enzyme that generates 1 µmol of NADH per min. LDH activity was normalized to control.

Caspase-3 activity assay. The activation of apoptosis was evaluated with the fluorescent substrate for activated caspase-3: Ac-DEVD-AMC (Enzo Life, ALX-260-031, Farmingdale, NY, USA). Ac-DEVD-AMC was prepared in 1000× in DMSO (20 mM). Cells were washed with cold PBS. Cold lysis buffer (50 mM HEPES, 1 mM EDTA, 0.1% Triton X-100, 5 mM DTT) was added to each sample. Cells were left at 4 °C for 15–30 min and cell lysate was collected and centrifugated at 12,000× *g* for 10 min at 4 °C. 10 µL cell lysate was then added to 90 µL cell lysis buffer containing Ac-DEVD-AMC (final concentration of 20 µM). Samples were incubated at 37 °C for 45 min. Fluorescence was measured at Ex/Em = 360/465 nm. For protein normalization, protein concentration of each sample was measured by BCA Assay (Millipore, Cat# 71285-3, Burlington, MA, USA). Caspase-3 activity was evaluated as previously described [49]. In brief, caspase-3 activity in each sample was calculated as follows:

$$Caspase\;Activity=\frac{{Fluor}_{Sample}{-Fluor}_{Background}}{\left[Protein\right](µg/µL)\times Sample\;Volume}$$

Arbitrary Units (A.U.) was defined as caspase activity normalized to control.

## Data Availability

All data used to prepare this manuscript are available in the main manuscript and in the Supplementary Materials.

## Supplementary Materials

The following supporting information can be downloaded at: https://www.mdpi.com/article/10.3390/molecules30153239/s1, Figure S1. Neither 5CadU nor 5CadC are incorporated into DNA. Figure S2. Hydrolysis of TFT to 5CadU at A) pH 7.4 and B) pH 8.0. Figure S3. Activation of necrosis in U87 cells treated with TFT and 5HmdU. Figure S4. Activation of apoptosis in U87 cells treated with TFT and 5HmdU. Figure S5. Nucleoside analogs are less toxic to normal human astrocytes (NHA).

## Abbreviations

The following abbreviations are used in this manuscript:

GBM	Glioblastoma
5HmdU	5-hydroxymethyl-2′-deoxyuridine
5HmdC	5-hydroxymethyl-2′-deoxycytidine
5CadU	5-carboxy-2′-deoxyuridine
5CadC	5-carboxy-2′-deoxycytidine
5BrdU	5-bromo-2′-deoxyuridine
TFT	Trifluorothymidine
