# Supplementary material for: Incorporation and Repair of Epigenetic Intermediates as Potential Chemotherapy Agents
Source: Molecules. 2025 Aug 1;30(15):3239. doi: 10.3390/molecules30153239 (PMC12348197; doi:10.3390/molecules30153239)
Supplement: Supplementary file 1 [file molecules-30-03239-s001.zip › molecules-3696876-supplementary.pdf]

# Incorporation and Repair of Epigenetic Intermediates as Potential Chemotherapy Agents

**Jason L. Herring [1], Mark L. Sowers [1,2], James W. Conrad [1], Linda C. Hackfeld [1], Bruce Chang-Gu [1,2], Rahul Dilawari [1] and Lawrence C. Sowers [1,3,*]**

[1] Department of Pharmacology and Toxicology, University of Texas Medical Branch, 301 University Boulevard, Galveston, TX 77555, USA
[2] MD-PhD Combined Degree Program, University of Texas Medical Branch, 301 University Boulevard, Galveston, TX 77555, USA
[3] Department of Internal Medicine, University of Texas Medical Branch, 301 University Boulevard, Galveston, TX 77555, USA
* Correspondence: lasowers@utmb.edu; Tel.: +1-409-772-9678

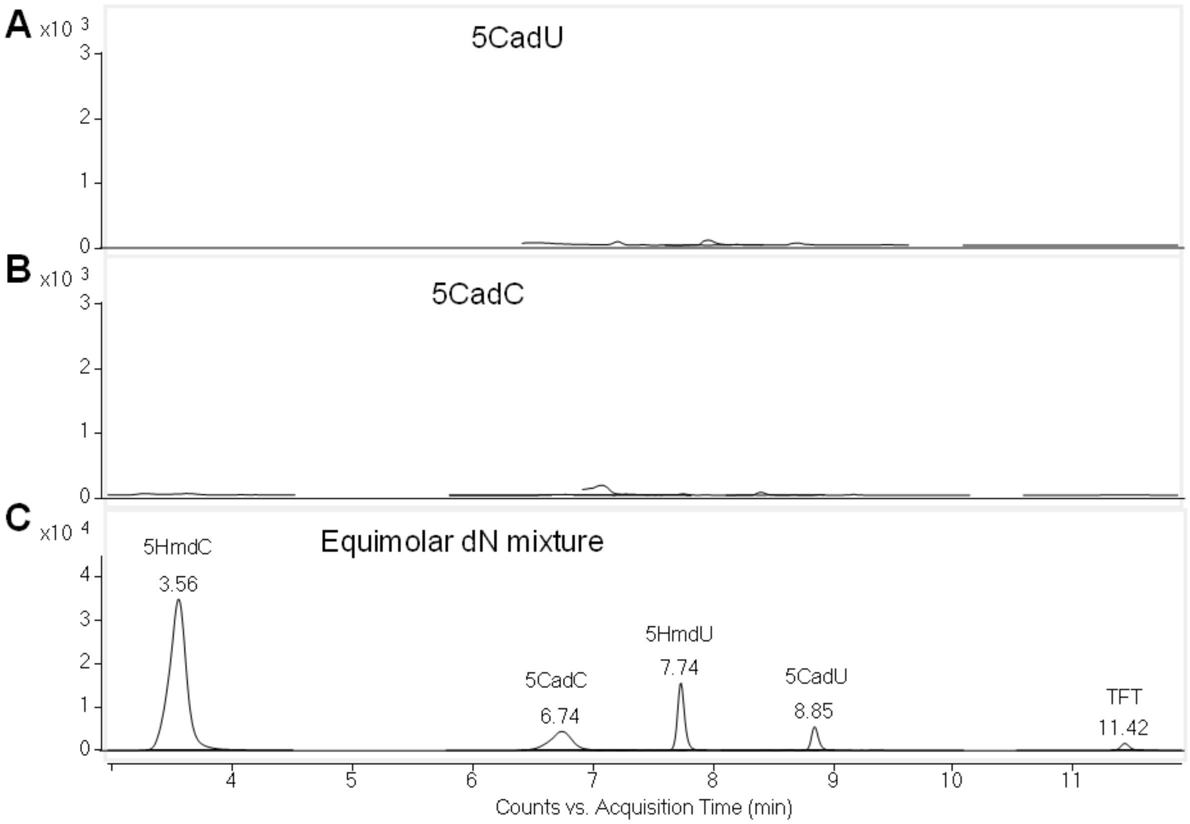


**Figure S1.** Neither 5CadU nor 5CadC are incorporated into DNA. A) DNA from cells incubated with 5CadU, B) DNA from cells incubated with 5CadC, C) Separation of an equimolar mixture of the deoxynucleosides by LC.

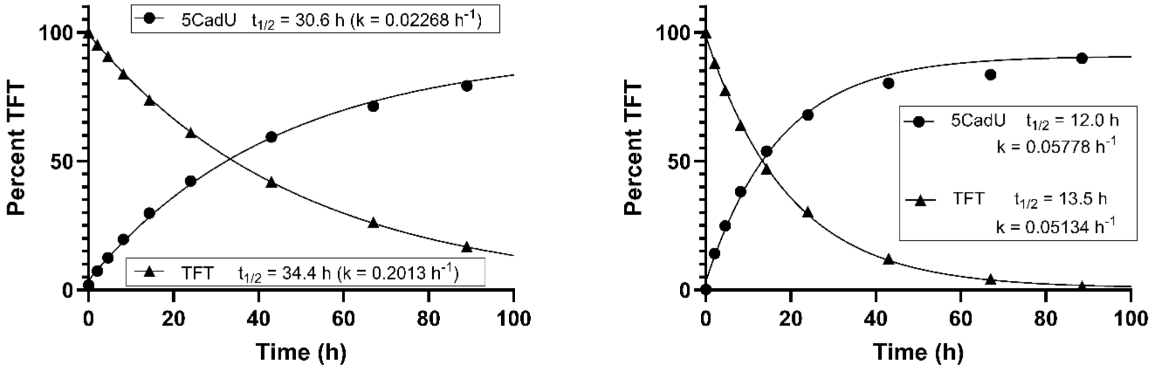


**Figure S2.** Hydrolysis of TFT to 5CadU at A) pH 7.4 and B) pH 8.0.

$$Y_{TFT} = Min + [(Y_0 - Min) \times e^{-kt}]$$

$$Y_{5CadU} = Max - [(Max - Y_0) \times e^{-kt}]$$

| | | t 1/2 (h) | k ($h^{-1}$) | $Y_0$ | Max | Min | $R^2$ |
|---|---|---|---|---|---|---|---|
| pH 7.4 | TFT | 34.4 | 0.02013 | 99.29 | | 0.17 | 0.9999 |
| | 5CadU | 30.6 | 0.02268 | 3.68 | 92.61 | | 0.9983 |
| pH 8.0 | TFT | 13.5 | 0.05134 | 98.61 | | 0.76 | 0.9995 |
| | 5CadU | 12.0 | 0.05778 | 3.19 | 90.75 | | 0.9896 |

**Rate constant equations and parameter fits were determined in Graphpad.**

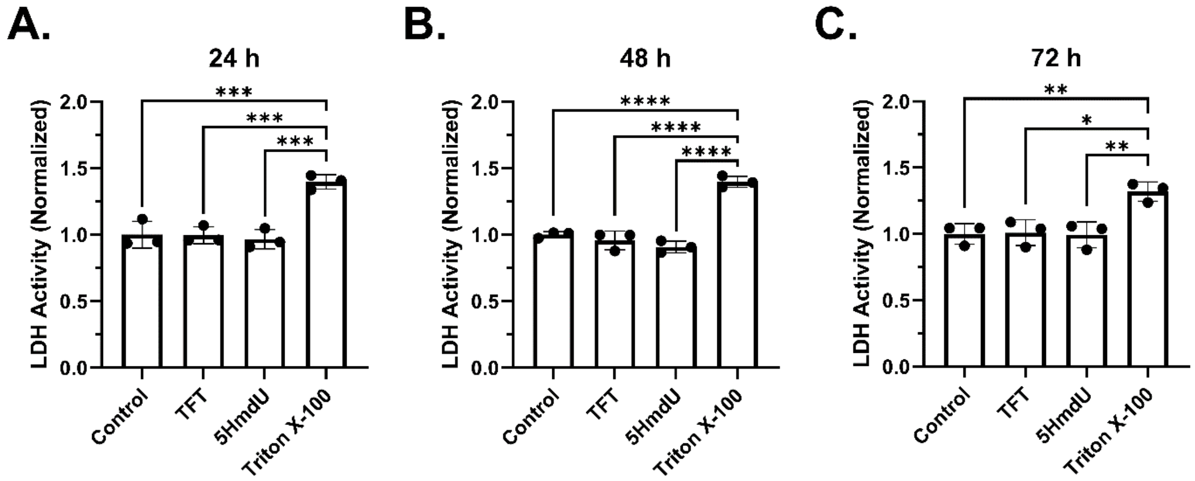


**Figure S3.** Activation of necrosis in U87 cells treated with TFT and 5HmdU. Early necrotic cell death was evaluated through detection of released LDH in media. 1 x $10^6$ cells were seeded into a 60 mm dish and treated with the 72 h $IC_{50}$ concentration of TFT (10 μM) and 5HmdU (48 μM). As positive control, 10% Triton X-100 was added in a separate well for 1 h. After exposure, media was collected for LDH activity at 24 h (A), 48 h (B), and 72 h (C). TFT and 5HmdU exposure do not increase LDH-activity at any time points. The average LDH activity and standard deviations were determined from biological triplicates. Statistical significance determined by ANOVA with post-hoc Tukey's test, *p<0.05, **p<0.01, ***p<0.001, ****p<0.0001.

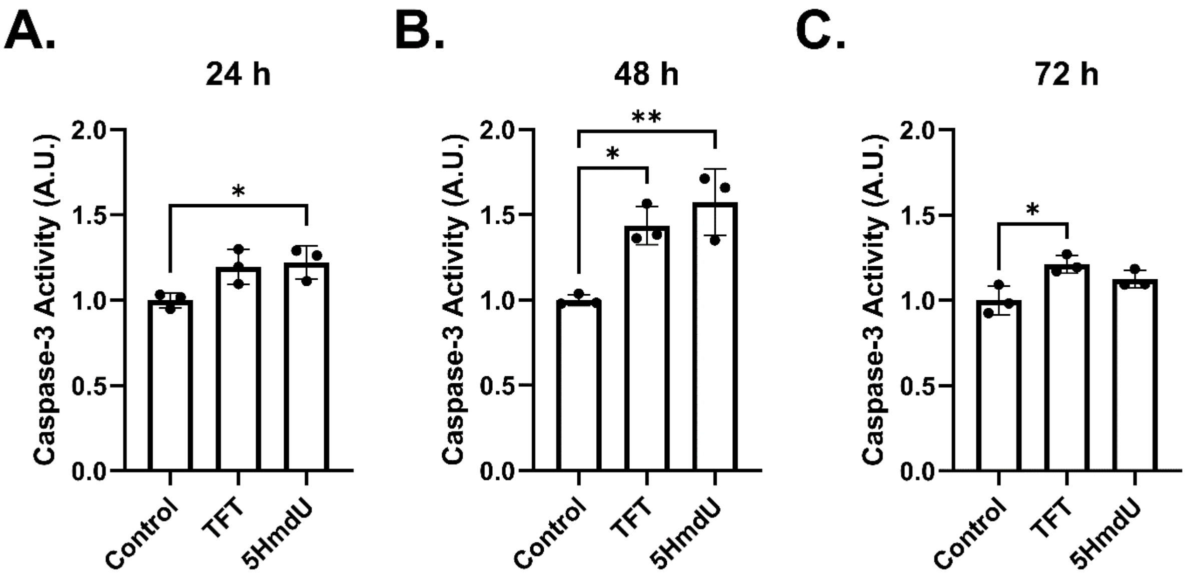


**Figure S4**. Activation of apoptosis in U87 cells treated with TFT and 5HmdU. Apoptosis activation was evaluated by measuring caspase-3 activity in cell-lysates of U87 cells treated with TFT and 5HmdU for 24 h. 1 x $10^6$ cells were seeded into a 60 mm dish and treated with the 72 h $IC_{50}$ values of TFT (10 μM) and 5HmdU (48 μM) for 24 h (**A**), 48 h (**B**), and 72 h (**C**). After exposure, cells were collected and lysed. Activity of caspase-3 in cell lysate was evaluated with the substrate Ac-DEVD-AMC and normalized to protein content**.** 5HmdU and TFT exposure significantly increases caspase-3 activity with the highest activity observed at 48 h after exposure. Arbitrary units (A.U.) were defined as caspase-3 activity relative to control. The average caspase-3 activity and standard deviations were determined from biological triplicates. Statistical significance determined by ANOVA with post-hoc Tukey's test, *$p<0.05$, **$p<0.01$.

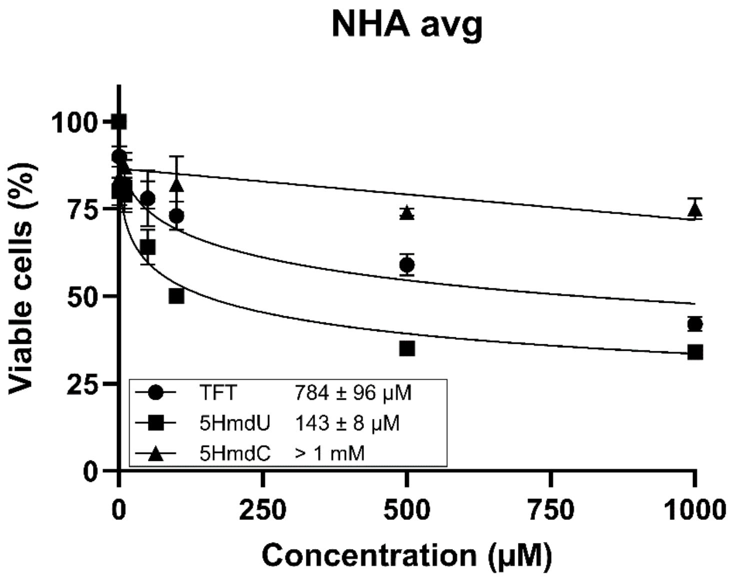


**Figure S5.** Nucleoside analogs are less toxic to normal human astrocytes (NHA). Approximately 2 x $10^3$ NHA in 100 µL DMEM were exposed to increasing concentrations of TFT, 5HmdU and 5HmdC nucleosides for 3 days in a humid 37 °C incubator with 10% $CO_2$ atmosphere. Cell viability was examined using the MTT assay after 3 days of exposure. MTT OD600 values were normalized to the untreated controls and plotted vs concentration. Average cell viability and standard deviations were determined from three experimental replicates.
